# Language Evolution: Why Hockett’s Design Features are a Non-Starter

**DOI:** 10.1007/s12304-014-9203-2

**Published:** 2014-07-19

**Authors:** Sławomir Wacewicz, Przemysław Żywiczyński

**Affiliations:** Center for Language Evolution Studies (CLES); Department of English, Nicolaus Copernicus University, Bojarskiego 1, Toruń, 87-100 Poland

**Keywords:** Evolutionary linguistics, Language origins, Biolinguistics, Animal communication, Displacement

## Abstract

The set of design features developed by Charles Hockett in the 1950s and 1960s remains probably the most influential means of juxtaposing animal communication with human language. However, the general theoretical perspective of Hockett is largely incompatible with that of modern language evolution research. Consequently, we argue that his classificatory system—while useful for some descriptive purposes—is of very limited use as a theoretical framework for evolutionary linguistics. We see this incompatibility as related to the ontology of language, i.e. deriving from Hockett’s interest in language as a product rather than a suite of sensorimotor, cognitive and social abilities that enable the use but also acquisition of language by biological creatures (the *faculty of language*). After a reconstruction of Hockett’s views on design features, we raise two criticisms: *focus on the means at the expense of content* and *focus on the code itself rather than the cognitive abilities of its users*. Finally, referring to empirical data, we illustrate some of the problems resulting from Hockett’s approach by addressing three specific points—namely *arbitrariness and semanticity*, *cultural transmission*, and *displacement*—and show how the change of perspective allows to overcome those difficulties.

## Introduction

It is hard to overestimate the impact of the *design features* model, proposed and developed by Charles Hockett in the 1950s and 1960s (1958; 1959, 1960a, [1960b] 1977; 1966; Hockett and Altmann 1968), which soon emerged as the default means of characterising animal communication systems and contrasting them with human language.^1^ More than a classificatory scheme, it became a reference point in more general considerations regarding the nature of human language, and has since heavily influenced linguistic courses and textbooks (cf. McGregor 2009; Yule 2010). Meanwhile, the development of cognitive science in the second half of the twentieth century (the “cognitive turn”, e.g. Bechtel et al. 1998) converged with the growing interest in evolutionary sciences (the “adaptive turn”, e.g. Gontier and Pina 2014) to yield an unprecedented upsurge of publications dealing with the evolutionary origins of language (Christiansen and Kirby 2003b). The point of view adopted in these texts was markedly different from the one inherent in Hockett’s system.

In this paper, we demonstrate that Hockett’s general theoretical perspective is largely incompatible with that of modern language evolution research, and that his classificatory system, while useful for some descriptive purposes, is of very limited use as a theoretical framework for evolutionary linguistics (and consequently, for the larger biosemiotic perspective). We see this incompatibility as deriving from Hockett’s interest in language as a product, leading to a “phenetic” classificatory system tracing superficial similarities. Specifically, we point to two underlying problems: *focus on the means at the expense of content* and *focus on the code itself rather than the cognitive abilities of its users*. We propose that the field of language evolution requires and presupposes a more “cladistic” approach to language: as a suite of sensorimotor, cognitive and social abilities that enable the use but also acquisition of language by biological creatures (the *faculty of language*, cf. e.g. Hauser et al. 2002; see also Wacewicz 2012). Such a stance leads to a more robust classificatory scheme that is open to extension into approaches that put a premium on the situated, social and distributed side of linguistic communication, and more general biologically grounded semiosis exceeding language (cf. “the biosemiotic turn”, Favareau 2008).

We begin by introducing Hockett’s system, and then contemporary research on language origins, with emphasis on the field of *language evolution* and the reasons behind the sudden surge of interest it has generated. Next we discuss two fundamental reasons why Hockett’s lists of design features cannot be integrated with this perspective. Those reasons are illustrated with three examples of this incommensurability: the features of *arbitrariness/semanticity*, *cultural transmission* and *displacement*, whose discussion we ground in recent empirical data. We conclude by proposing an alternative approach to (what we take to have been) Hockett’s primary goal, i.e. capturing the difference between the communication of humans and non-human animals.

## Hockett: Language and its Design Features

Hockett’s reflection on the design features of language can be divided into three phases: the initial statement (1958 and 1959), which explains a comparative and cumulative approach to defining language; the best known presentation from “The Origin of Speech” (1960a) and the most extensive one from “Logical considerations in the study of animal communication” ([1960b] 1977), where Hockett enumerates thirteen design properties and proceeds to discuss them in an evolutionary framework; and later presentations (1966; 1968) with the most extensive list of sixteen design features, in which his attention shifts from comparative concerns to systemic properties of language.

### The Original Proposal

Hockett first discussed the design features of language in *A Course in Modern Linguistics*, a linguistics textbook for college students. (Hockett [1958] 1967). Although Hockett adopts there the view that linguistics is an autonomous field of knowledge,^2^ he also shows a distinct naturalistic sentiment. This is evident for example in his use of the biological terms “ontogeny” and “phylogeny” with reference to language acquisition (1958: 353ff) and historical language development respectively (1958: 353ff), or an accentuated claim that there must be a genetic component to human language (1958: 353–354). But Hockett’s naturalism is most often indicated by his insistence in viewing language as behaviour—or rather a system which manifests itself in linguistic behaviours—accompanied by the methodological postulate that the study of language should be the study of such observable linguistic behaviours (see e.g. 1958: 137–144, 322). Clearly, the concept of “habit” betrays an influence of behaviouristic psychology. However, Hockett’s emphasis that language should primarily be understood as a set of behaviours makes his description more akin to ethology than psychology. Given such an attitude, it comes as no surprise that he is interested in comparing linguistic behaviours with other communicative behaviours, including communicative behaviours of non-human animals. These comparative remarks are presented in the postscript to the book (section 64), entitled “Man’s Place in Nature” (1958: 569–586), which constitutes the first exposition of “design features of language.”

At this juncture, it should be noted that Hockett adheres to the traditional, *code* model of communication (see Shannon 1948), where communication is understood as *transmission of information* from the sender to the receiver (allowing the former to impact the latter’s behaviour). In this particular respect, Hockett’s view aligns with the accounts of communication found in biological sciences, e.g. sociobiology (see Wilson 1975), ethology (Hailman 1977), or behavioural ecology (Krebs and Dawkins 1984).^3^ Rather than provide strictly definitional criteria for communication and language, Hockett opts for a more heuristic approach. He compares and contrasts selected properties of language with properties of selected non-human communication systems—bee dancing (Frisch 1950; Carpenter 1940), stickleback courtship (Tinbergen 1953), herring gull care of offspring (Tinbergen 1953), and gibbon calls (Carpenter 1940), which he knew of from the ethological literature of his day. He also looks at selected human non-linguistic communication codes—the Morse Code and the Ogam script used by speakers of Old Irish.^4^

In *A Course in Modern Linguistics*, Hockett doesn’t refer to these properties as “design features of language” but calls them “the key properties of language”. He enumerates seven of them: *duality*, *productivity*, *arbitrariness*, *interchangeability*, *specialisation*, *displacement* and *cultural transmission* (1958: 574). Hockett refrains from qualifying the seven properties as more or less important but seems to treat them as equally fundamental to the characterisation of language. For comparative purposes, Hockett uses the terms *ceneme* and *plereme* borrowed from Hjelmslev’s linguistic theory,^5^ when introducing the feature of duality and comparing it to other means of communication, i.e. Morse code and the Ogam script (1958: 574–575). Accordingly, a communicative system possesses duality if it consists of the cenematic plane, comprising differential, signalling units (such as phonemes in language, or dots and dashes in the Morse code), and the plerematic plane, which contains units of expression with meaningful content (such as morphemes in language or Morse code combinations of dots and dashes) (Hockett 1958: 575).

The discussion of the seven properties of language opens with a comparative chart that illustrates how, in Hockett’s opinion, each of them turns up or fails to turn up in four non-human systems of communication—bee dancing, stickleback courtship, herring gull care of offspring and gibbon calls^6^ (1958: 574). In this presentation and later ones (most importantly in “The Origin of Speech” from 1960; see below), Hockett attributes duality to none of the non-human systems of communication he describes, although he doesn’t exclude the possibility that some forms of non-human communication may actually possess it (Hockett 1958: 575). Next, *productivity* is defined—rather predictably—as the ability, gained by a child during the process of linguistic ontogenesis, to produce novel utterances (1958: 575–576). Hockett explains that productivity of this sort is possible through combining or “blending” simple pleremes into complex ones, and insists that, apart from human communication, it characterises honeybee waggle dance, where a worker bee “can report on an entirely new source of nectar” (1958: 577). Later, he discusses *arbitrariness* in relation to the iconic character of bee dancing, whose moves stand for the direction and distance to a source of nectar. By way of contrast, a string of phonemes doesn’t bear any resemblance to the meaning associated with this sequence in a language. The property of *interchangeability* consists in alternating the sender-receiver roles in the way that is typical of conversational interaction. Hockett acknowledges that this feature is present in bee dancing and gibbon calls but denies its existence in other non-human communication systems known to him (Hockett 1958: 578).

A more involved explanation is offered with regard to *specialisation*. Hockett first defines communication in general terms as a process whereby one organism takes an action that triggers a behaviour in another organism (1958: 578). To determine the extent to which a communicative system is specialised, its trigger conditions and the direct physical consequences of a message must be compared—if they are closely related, a system is not specialised; if, on the other hand, there is no direct link between them, it is specialised. Language is an example of a highly specialised system of communication, because the sound waves produced by speaking are not rigidly linked to the hearer’s behaviours.

The explanation of *displacement* rests on the notions of antecedents (verbal messages) and consequences (behaviours caused by messages): “A message is displaced to the extent that the key features in its antecedents and consequences are removed from the time and place of transmission” (1958: 579). Language possesses the property of displacement due to the fact that verbal messages can refer outside the spatial and temporal context of their production, and likewise can induce behaviours outside this context.

The section devoted to *cultural transmission* opens with the identification of two mechanisms responsible for establishing the conventions of a communicative system within a particular organism: one is that of genetic inheritance, the other of cultural transmission (1958: 579). Cultural transmission is defined by Hockett as involving learning—such as a child learning a language or a rat learning to run a maze—and teaching, characterised by the transmission of a behaviour from one organism to another by physical demonstration (1958: 579). He takes the view that the conventions of language are transmitted culturally, rather than genetically, and that no non-human communicative system that he is familiar with involves cultural transmission (1958: 580).

### “The Origin of Speech” and “Logical Considerations”

After *A Course in Modern Linguistics*, Hockett spent several years investigating the definitional criteria for language and published several papers on it. The first was “Animal ‘Languages’ and Human Language” (1959), in which he repeated his previous arguments about the seven properties of language. “The Origin of Speech”, a *Scientific American* contribution (1960a), and “Logical considerations in the study of animal communication” (1960b)^7^ saw the extension of the list and a deepening of the comparative mode of reflection, which Hockett described as the “method modeled on that of the zoologist” and whose frame of reference is such that “all languages look alike when viewed through it, but … within it human language as a whole can be compared with the communicative systems of other animals, especially the other hominoids, man’s closest relatives, the gibbons and great apes” (1960a: 5). It is also there that he elaborated on the idea of “design features,” i.e. features shared by all human languages, some of which may appear “trivial” but “become worthy of mention only when it is realized that certain animal systems—and certain human systems other than language—lack them” (1960a: 6). Hockett presented a list of 13 design features, which included the seven properties he identified previously.^8^ To these he added *vocal-auditory channel*, *broadcast transmission and directional reception*, *rapid fading*, *total feedback*, *semanticity*, and *discreteness*.

With regard to the *vocal-auditory channel* feature, Hockett observes that “The signals used in any language consist … of patterns of sounds, produced by motions of the respiratory and upper alimentary tract” (1960b: 126). The definition of the channel feature is appended with an observation that the ability to control vocalisations in humans (e.g. to use vowel colour distinctively) stems from the cortical control of speech (1960b: 127–128). Addressing evolutionary concerns, he offers a rather simplistic comment that the primary advantage of the vocal-auditory channel consists in leaving “much of the body free for other activities that can be carried out at the same time”^9^ (1960a: 6) or leaving “hand and eye for other purposes” (1960b: 129)

*Broadcast transmission*/*directional reception* and *rapid fading* are presented as directly stemming from the properties of the channel. The first of these refers to the fact that a linguistic signal can be received by any auditory system within earshot, while its origin can be traced back to a particular location (by means of binaural direction finding) (Hockett 1960a: 6, 1960b: 131–132). *Rapid fading* describes the instantaneous disappearance of language utterances, which is unlike more permanent signals and signs, such as animal tracks, but similar to animal warning calls and other vocalisations (Hockett 1960a: 6, 1960b: 133–134). In Hockett’s view, redundancy characteristic of linguistic communication is an effect of the transitory nature of speech (1960b: 134).

The *total feedback* of language, Hockett argues, means that the speaker hears everything she says. This is unlike body signals that rely on the visual channel, where the sender may not be able to see their own signals. For instance, in the stickleback courtship the male cannot see the colours of its own belly and eyes, even though these are crucial for stimulating the female. (1960a: 6, 1960b: 135). The *semantic* property of language is explained as depending on fixed associations between elements in a message and recurrent features and situations in the world. Hockett opts for a liberal understanding of semanticity, arguing that whenever a communicative behaviour is tied in a fixed way to appropriate elements of the environment, such a behaviour should be classed as semantic; accordingly, gibbon food calls and a rate or direction of bee dance are taken by him to be semantic (1960b: 142). Finally, *discreteness*—referring to the absolute functional distinctiveness of linguistic signalling units—is contrasted with the analog, or scalar, nature of both vocal gestures, e.g. a cry of anger, and the moves of bee dancing (Hockett 1960: 6).

The introduction of the six new properties doesn’t alter Hockett’s conception of language and the way he defines it. In fact, all of them can be deduced from the old set of features. In the previous format comprised of the seven features, Hockett insists that linguistic behaviour prototypically manifests itself in the vocal auditory channel and—as already indicated—broadcast transmission/directional reception, rapid fading and to an important extent total feedback describe selected characteristics of this channel; whereas the features of semanticity and discreteness were previously subsumed under the discussion of duality. Even in “Logical considerations” (1960b), which contains the most extensive exposition of the design features ever offered by Hockett, the comparative notes are grossly underdeveloped—he does not really compare language to, say, the gibbon song call system, bee dancing or stickleback courtship ritual but rather points to local similarities and contrasts between these when presenting the respective design features.

### Later Presentations

The format of 13 properties is—in the tertiary literature on language and linguistics—treated as the standard presentation of Hockett’s design features (see e.g. Crystal 1987: 396–367; Hauser 1996: 47–48). In later accounts, “The Problem of Universals in Language” (Hockett 1966) and “A Note on Design Features” (Hockett and Altmann 1968), he concentrates on the properties of language itself. These considerations lead him to posit three additional properties—*prevarication*, *reflexiveness* and *learnability*—giving in total a list of sixteen features. In the 1966 account, Hockett uses the concept of design features as a platform to discuss language universals that pertain to extremely versatile properties of language, ranging from very general ones, such as the existence of a language in every human culture or the primacy of spoken language over its written form, to specific aspects of grammatical description, such as the presence of proper nouns in every language or the universality of distinctions in vowel quality.

Out of the three newly introduced design features, only *learnability*, which refers to the fact that speakers of a language can learn a new language, is truly innovative—with regard to prevarication and reflexiveness, Hockett demonstrates how they result from the previously discussed properties. Thus, *prevarication*, understood as the capacity of linguistic messages to be false or meaningless in the logical sense, depends on semanticity, without which a message couldn’t be tested for validity or meaningfulness at all. It also depends on displacement, which seems to be a precondition for a successful lie, and openness, which in turn guarantees the possibility of generating new, i.e. also meaningless, messages. The property of openness is also vital to the definition of *reflexivity*, whereby language allows its users to communicate about communication—Hockett notes that in an extremely open code, such as language, new meanings are easily attached to either new or old elements, giving this type of system the potential to communicate about anything, including reflexive communication about itself.

### Significance

As already indicated, Hockett’s concept of design features has dominated linguists’ thinking about language origins and language in relation to other communicative systems, which is probably best reflected in linguistics textbooks (e.g. McGregor 2009; Yule 2010). While linguists as well as other scholars have routinely drawn on individual features, there has been surprisingly little targeted, critical discussion of the system as a whole (but see Hauser 1996). To a considerable extent, this frame of thought has been inherited by language evolution literature, where it often remains influential as a starting point, inspiration, or conceptual base (see e.g. Aitchison 2007; Fitch 2010).

But Hockett’s system has had much wider influence. For example, ethologists implemented the design-feature approach to the study of selected non-human communication systems, particularly in the 70s of the last century (e.g. Marler 1970; Thorpe 1972; Hinde 1975). In general semiotic and biosemiotic literatures, Hockett’s classification appears frequently as a foundational attempt to systematically differentiate between human and non-human communication (see e.g. Danesi and Perron 1999: 109–111; Martinelli 2010: 221; Nöth 1990: 155–156).

## Evolution of Language—a Recent Perspective

*Evolution of language* (or: *language evolution*) is best described as a research area unified by a common goal: to explain the emergence and subsequent development of the species-specific ability of human beings to acquire and use language. It should be distinguished from both historical linguistics and a narrower notion of the evolution of languages (plural), the latter being a quasi-evolutionary, long term historical change in modern-day linguistic systems (see Hurford 1999). Short introductory texts include papers by Christiansen and Kirby (2003a), Fitch (2002), and Hurford (2003); more recently, a handbook (Tallerman and Gibson 2011), as well as textbooks (Johansson 2005), and monographs (Fitch 2010) have become available.

Language evolution is a continuation of the inquiries launched by former generations of philosophers and philologists, aimed at explaining the origin of language. Nonetheless, *the raison d’être* of the field is making itself qualitatively different from all such previous attempts: by its drawing on interdisciplinary empirical research, its fully naturalistic, biologically-oriented framework, its increasing reliance on formalism, and its focus, for the most part, on the cognitive side of language use. As such, it is a relatively recent perspective that has nevertheless gained considerable momentum over the last two decades (possible to measure quantitatively, see e.g. Christiansen and Kirby 2003b).

Contrary to some commentators (e.g. Gong et al. 2014), research on language origins was not nearly absent between the famous 1866 “ban” of the Linguistic Society of Paris and the 1990s. Gordon Hewes (one of the pioneers of modern-style language evolution research and a proponent of an early version of the gesture-first hypothesis) lists ten or so works related to language origins for every intervening decade (Hewes 1996). The symbolic caesura is often put at 1990, with the influential paper by Pinker and Bloom (1990). In 1991, Kendon symptomatically states:

Discussion of the problem of language origins has by now become quite widespread and certainly highly informed. It may still not be fully respectable; and many still regard it as, at best, a kind of intellectual game. If this is what it is, it is nevertheless a much more interesting and challenging game than it once was, and it provides a focus through which a wide range of highly diverse fields of knowledge and theory may be brought into relationship with one another. (Kendon 1991: 202)

Why, then, was it the 1990s that saw the breakthrough? Before we have mentioned the “cognitive turn” and the “adaptive turn”, which we may call the *Chomskyan factor* and the *Kuhnian factor*, but they were complemented by the *empirical factor*. The qualitative transition from the “intellectual game” of guessing and telling “just-so stories” to a more scientific enterprise was only made possible by major advances in the availability of empirical data bearing on the question of language origins. The main contributing disciplines have been comparative studies on animal communication (e.g. Arnold and Zuberbühler 2006; Hauser 1996), animal cognition (e.g. Griffin 1992), neurosciences (e.g. mirror neurons, Rizzolatti et al. 1996), speech physiology (Fitch 2000), genetics (e.g. Enard et al. 2002), mathematical and computational modelling (e.g. Nowak et al. 2001), experimental psychology (e.g. Kirby et al. 2008), gesturology (e.g. McNeill 2005) and sign language studies (e.g. Emmorey 2002), and paleoanthropology (e.g. Wilkins and Wakefield 1995) and archaeology (e.g. McBrearty and Brooks 2000).

It is worth noting that language evolution research continues to change dynamically. Traditionally, a majority view in the field has been that “language evolved from animal cognition, not from animal communication” (Ulbaek 1998: 33), through gradualistic Darwinian selection (Pinker and Bloom 1990). However, recent research has led to important revisions, extensions, or even challenges to that dominant position (Dor and Jablonka 2014). For example, attention to factors such as multilevel selection, niche construction or epigenetic inheritance has played an increasing role in enhancing the Darwinian paradigm in the spirit of the extended synthesis (cf. Pigliucci 2009). Also the role of culture and cultural evolution has been of growing importance within language evolution studies (e.g. Kirby et al. 2008). Critical to those debates is the foundational question of the nature of *language*: while influential scholars have argued for a very narrow delineation of this term (Hauser et al. 2002), most those in the field see language as a complex (or *mosaic*—Hurford 2003) of cognitive skills, or an even more multifaceted phenomenon, grounded in but transcending individual cognition (e.g. Gärdenfors 2004). All of this shows promise for the integration of the language evolution research within larger scale theoretical frameworks (cf. e.g. Barbieri 2010).

## Criticisms of Hockett

It may seem that the standpoint of Hockett differs from that of language evolution principally in focusing on actual, existing systems as opposed to explaining continuity and descent; in reality, the conflict is much more fundamental. As we explain below, it results from profound differences in the assumed perspectives on what language is and what aspects of it are theoretically interesting. Here, we single out the criticisms against Hockett’s system that we consider particularly telling as to why it gets stuck on surface similarities and effectively fails to capture all the relevant ways in which language truly differs qualitatively from other kinds of animal communication. These shortcomings, as viewed from the perspective of language evolution, hinge upon two (closely related, but distinct) major issues: firstly, Hockett’s classificatory system *focuses on the means*, especially the physical properties of the medium of transmission, at the expense of content, and secondly, it *focuses on the code itself*, rather than the cognitive abilities of its users that make language use possible in the first place. Let us consider these reservations in turn.

### Focus on the Means at the Expense of the Content

It has been observed (e.g. Lyons 1998: 146) that Hockett’s system shows a clear, and explicitly stated, bias towards oral over gestural and other types of linguistic communication. This is reflected by the first five features from “The Origin of Speech” (1960a): Vocal-Auditory Channel, Broadcast Transmission and Directional Reception, Rapid Fading, Interchangeability, Total Feedback. Except Interchangeability, all of them directly or at least very closely concern the properties of the vocal/auditory modality. This, in turn, stems from a deeper problem, namely the focus placed on the form/structure rather than the content/function.

Favouring speech over its alternative(s) is unsubstantiated in three related ways. Firstly, as is now well known, sign languages are fully equivalent to spoken ones in practically every relevant respect, including morphosyntax, dialectisation, historical change, rough cerebral localisation, and acquisition by children (see Petitto 1994; Emmorey 2002). Secondly, language is largely modality independent, in that even if speech or sign are granted a certain special, primary status, the actual communication acts can be carried out exploiting other channels (cf. writing, the Tadoma method, or to some extent whistled languages). Thirdly, under normal circumstances, natural conversation is never unimodal, but rather multimodal (Kendon 2004), with co-speech gesture, and even full body movement and facial expression being parts of the complex message and complementing it with nonredundant and communicatively important semantic information (Goldin-Meadow 2011). This, of course, can be further extended by abandoning the code model of language altogether in favour of its alternatives, e.g. the distributed approach (e.g. Rączaszek-Leonardi 2009, 2012), on which language does not constitutively depend on any specific modality or means of transfer, but rather results from a network of social practices as a means of their coordination.

Favouring speech over its alternative(s), far from remaining neutral, has ramifications for other aspects of how we define language. In the evolutionary context, it gives rise to two misconceptions. Firstly, it introduces an unfounded bias against looking for the origins of language in gestural communication. The gestural approach to language evolution inaugurated by Gordon Hewes (1973), while not without its critics (e.g. Tallerman 2011), has been increasingly influential and has become an extensive and vigorously explored research area in its own right; currently different versions of gesture-first theories (e.g. Corballis 2002; Arbib 2005; Armstrong and Wilcox 2007; see also Donald 1991; Zlatev 2008) are serious contenders in the field. Secondly, the oral/vocal bias promotes an equally unfounded assumption of continuity between language and extant vocal communication of non-human primates. Human language is qualitatively different from primate vocal communication and it is not clear whether it evolved “from” it in any interesting sense beyond the obvious anatomical substrates (“Focus on the Code Itself, Rather than Cognitive Abilities of Its Users” Section). Note that this is logically independent of the question of gestural primacy. Even if we assume uniform evolution of language in the vocal modality, language requires development of novel cognitive and neural mechanisms that are largely separate from those underlying e.g. alarm calls (see “Example: arbitrariness and semanticity” Section).^10^

Thus, focus on the physical characteristics of the medium of signalling is misplaced. Hockett’s features mentioned above may be useful descriptively in capturing interesting facts about speech (e.g. how rapidity of fading relates to duality of patterning, Galantucci et al. 2010), but they tell us next to nothing about the qualitative difference between language and other communicative systems.

### Focus on the Code Itself, Rather than Cognitive Abilities of Its Users

Undoubtedly, most or all of the features in the second part of Hockett’s list as stated in (Hockett and Altmann 1968: 63–64), and particularly *arbitrariness*, *displacement*, and *prevarication*, are highly relevant to the perspective of language evolution. But when understood as the properties of the code, they have very limited explanatory value. Neither the analysis of the structural properties of individual utterances, nor of the structural properties of the entire abstract system is capable of explaining to any interesting degree how it is possible for agents to establish unmotivated conventions, to denote entities that are spatiotemporally absent from the immediate surroundings, and to intentionally convey false information. The existence of such properties in the code is possible only epiphenomenally, as a function of the cognitive-representational abilities of the users of the code. The required shift could start from a ‘move inwards’, that is refocusing from animal communication to animal cognition; this reflects a transition from a phenetic classificatory approach, which traces surface similarities, towards a more cladistics one, which is oriented to deeper-level mechanisms.

In the context of evolutionary study, this change has a vital corollary, namely a profound redefinition of evolutionary continuities and discontinuities in the emergence and development of language. According to a once popular belief, language might have arisen from animal calls becoming gradually more structurally complex (e.g. Hockett 1958: 582). Now we know this conjecture to be false. Human language and the communication of (nonhuman) animals operate according to different principles and the gap between them cannot be bridged by reference to any modifications of the communicative medium alone, whether selectionist or chance (see e.g. Deacon 1997). No increase in structural complexity can in and of itself suffice to explain this transition without considering the underlying “machinery”: broader-scale cognitive abilities such as cooperation with non-relatives, shared intentionality, metarepresentation and Theory of Mind, mimesis and intentional imitation, enhanced memory and executive function, symbolic representation, open-endedness, and recursion (cf. Deacon 1997, 2011; Donald 1991, 1999; Hurford 2003; Tomasello 2008).

From the phylogenetic perspective of language evolution, directing attention towards the communicative code itself (and neglecting the mechanisms underlying its production and reception) creates puzzling continuities, such as between humans and bees (see footnote 13), monkeys or gibbons (cf. Hockett 1960a: 10–11). Below, we exemplify how many of those problems disappear and the expected (human—great ape) continuities reappear when we turn our attention away from communication towards general cognition; and even within communication—away from the modality towards the content.

A note is in order. In line with the majority view in language evolution, our text explicitly prioritises the “individualistic-internalistic” perspective. As we stated in section “Evolution of language – a recent perspective”, this should not be treated as exclusionary to other perspectives, but rather as a first step or starting point, open to enhancing with socially and ecologically oriented views, and in particular those treating language as a collective (cultural) invention. An interesting example can be found in Hurford (2008), who in his summary of the differences between language and animal communication alongside cognitive traits such as “mindreading” lists systemic traits such as “diversity” or “self-organisation”.

#### Example: Arbitrariness and Semanticity

Arguably the most widely discussed phenomenon in animal communication, vervet monkey alarm calls have captured the attention (and, it seems, imagination) of numerous authors after Hockett. As is well known, the calls of vervet monkeys demonstrate a kind of referential specificity, termed *functional reference* (e.g. Hauser 1998)—in that each of them “denotes” a separate class of predators (where “denotes” means it is reliably produced as a response to the right stimulus on the one hand, and reliably triggers the appropriate escape strategy). Since the calls can be interpreted as being “about” certain creatures, and since their acoustic structure does not resemble the “referent” in any way, they can be ascribed *semanticity* and *arbitrariness*. This paves the way for an inflationary interpretation of vervet monkey alarm calls, with many researchers inclined to see them as a kind of proto-symbols, proto-names, possibly not unlike first words (e.g. Diamond 1992; Leakey 1994; Dunbar 1996; Maynard Smith and Szathmáry 1999; Aitchison 2000; Kurcz 2000; Calvin and Bickerton 2001).

This inflationary approach is clearly mistaken. Alarm calls do exhibit other interesting properties, e.g. audience effects or potential for (semantically noncompositional) productivity (see e.g. Slocombe 2011), which prevent their dismissal as rigid, mechanistic stimulus–response patterns. However, alarm calls exist in small innate inventories that cannot be expanded, the calls themselves are nonarbitrary and have a largely fixed innate structure, they are semantically noncompositional, they are only partly voluntary, and they are controlled by the limbic areas of the brain rather than the neocortical areas (Deacon 1997: 54–59, 234–235). Vervet monkey alarm calls are fairly rigidly coupled with corresponding escape strategies, their apparent semanticity arising more from ecological constraints than some deeper cognitive insight (i.e. diversified alarm calls tend to be absent in species that employ a uniform escape strategy against all predators; see Manser et al. 2002). In short, monkey alarm calls and words are only superficially alike, while being *unlike* each other in most relevant respects. Finally, alarm calls are present in a number of nonprimate or even non-mammalian species (e.g. chickens, Evans et al. 1993).

The clearest case of arbitrariness and semanticity manifested by a non-human comes, not surprisingly, from the apes and from visual rather than vocal communication—the behaviour in question is the use of lexigrams by enculturated apes such as Kanzi (Savage-Rumbaugh and Lewin 1994). But an interesting phenomenon is reported in wild apes by Savage-Rumbaugh: bonobo troops during migration seem to purposefully leave branches at path crossings, possibly to indicate travel directions to other troop members. Zlatev (personal communication) points to the fact that, if supported by better documentation, this phenomenon would count as significational (representational via signs) and intentional (both as “being about” and “voluntary”).

Apes can easily complete tasks requiring processing of arbitrary tokens, even ones involving a certain level of abstraction, e.g. correctly identifying relations between relations, such as “same” or “different” (Thompson et al. 1997). Although similar results have been reported with monkeys (Fagot and Thompson 2011), they were achieved after thousands of acquisition trials, suggesting a different underlying cognitive mechanism.

#### Example: Cultural Transmission

In Hockett (1958: 580, 1960a: 6), the calls of gibbons were credited with some potential for cultural transmission, a feature that was later changed to *tradition* as qualifying requirements for cultural transmission were revised (Hockett 1960: 6). Vocal learning in general is an important feature of human language, and research on other vocal learners, such as songbirds (Fehér et al. 2009), sea mammals (Janik et al. 2006), or even the limited degree of vocal learning that is exhibited by the great apes (e.g. Taglialatela et al. 2012) is of course immensely relevant to language evolution. For example, comparative research offers vistas into such areas as windows/critical periods in the acquisition of vocalisations (Marler and Peters 1987), relaxation of selection pressures (Takahasi and Okanoya 2010), homologies in the anatomical and neural control of vocalisations (Ghazanfar and Hauser 1999), and even “deep homology” in their genetic underpinnings (Fitch 2010: 55–57). The problem with cultural/traditional transmission so conceived is that, again, it has to do purely with the properties of the medium, i.e. the vocal patterns. This is only superficially, if at all, related to what truly counts about human cultural transmission. The qualitative difference setting off human from animal communication systems is the social transmission (vertical *as well as horizontal*, i.e. within and between groups and generations) of intersubjective conceptual *contents*, i.e. of semantic information. This mechanism enables the so-called “ratchet effect” (Tomasello 1999)—the preservation and incremental build-up of knowledge across generations, giving rise to technological progress among other things characteristic of human-style cultures.

Interestingly, Hockett does mention proto-cultural phenomena in chimpanzees, but in a rather dismissive spirit (cf. the single sentence in Hockett (1960b: 157) “[s]ome short-lived traditions have been observed among chimpanzees in captivity (*fide* Spuhler)”). But once again, when we shift the perspective away from communication, we find clear patterns of group-specific, culturally transmitted behaviours in chimpanzees: “chimpanzee cultures” have been widely recognised as real at least since the influential paper by Whiten et al. (1999). Here, as in other cases, cognition is crucial. The behaviours themselves are interesting but superficial manifestations of the underlying capacity for culture, and it is this underlying “social-cognitive, social-motivational infrastructure” (Tomasello 2008) that should be the real focus of attention.

#### Example: Displacement

The celebrated example of the bees showcases another weakness to which Hockett’s system proves to be vulnerable. Many (e.g. Kurcz 2000: 30) have noted that the unusually high rating of bee dance among animal communication systems is not substantiated on any independent grounds, especially given the phylogenetic distance of our clades. Hockett himself did not overlook this issue, commenting on the dance being limited only to one thematic variable (location of nectar), which considerably weakens the comparison to human language (1958: 571; see also “The original proposal” Section). But such a statement is an ad hoc explanation, betraying a conceptual hole in the network of distinctions.^11^

Displacement had an important place on Hockett's list, and is still considered a pivotal skill by leading language evolution researchers (e.g. Deacon 2011; Hurford 2011; Tallerman 2011). However, the interest of language evolution lies in displacement as a cognitive capacity rather than an externalised feature of the communicative code. So far there is no evidence of displacement in the communication of non-human great apes. However, this case presents a particularly severe “absence of evidence versus evidence of absence” problem because of methodological difficulties: the spatial and/or temporal distance constitutive of displacement prevents drawing inferences about displaced messages. Once again, what evidence we do find of displacement is not in the sphere of communication, but rather of cognition. Clearest examples come from research on food caching species, e.g. corvids, where in addition to impressive spatial memory for locations of stored food there is some evidence for episodic-like memory and advanced strategies for recovery and cache protection (e.g. Emery and Clayton 2001). More generalised foresight and future planning may be specific to the great ape species. For example, Gomes and Boesch (2009) report a long-term (but not short-term) tendency of females in a wild chimpanzee population to preferentially mate with males who have shared meat with them. More robustly, Osvath and Osvath (2008) experimentally demonstrated forethought and future planning in captive chimpanzees and an orangutan, who were shown to forfeit a smaller food reward in favour of a tool that they could use—later and in a different location—to retrieve a larger food reward. Another example involves deliberately caching projectiles for further use by a captive chimpanzee (Osvath 2009).

## Conclusions

Hockett’s system of design features, although still potentially valuable for other purposes, is radically unfit for capturing the difference between the communication of human and non-human animals from an evolutionary perspective, and thus it cannot be fruitfully integrated into the larger framework of studies within this perspective. The reasons for this fundamental incompatibility lie chiefly in the misplaced interest in the structure and medium of the communicative signal, while it should be placed on its content, the minds of its users, and the social and ecological context of use.

In contrast, language evolution needs a primarily “internalistic” perspective, directly informed by modern evolutionary theory and social and ecological perspectives. An idealised “complete” evolutionary explanation for language would require an “LCA baseline” (socio-cognitive and anatomical skills of the last common ancestor of *Homo* and *Pan*), a chronology of stepping stones (further development of those skills), and a plausible but falsifiable scenario of selection pressures that would have led to the achievement of those stepping stones. Crucial to the explanation are the social, cognitive and anatomical preadaptations (stepping stones) that are not directly visible in communication but are the necessary prerequisites. As mentioned above, the main areas whose investigation shows most promise for a better understanding of the sources and character of this uniqueness of language are: cooperation with non-relatives, shared intentionality, metarepresentation and Theory of Mind, mimesis and intentional imitation, enhanced memory and executive function, symbolic representation, open-endedness, and recursion—all of which are better understood as cognitive skills rather than features of language in the E-sense.

This paper should not be treated as a comprehensive evaluation of Hockett’s design features. For example, we do not question the descriptive value of his system, nor do we deny the productive applicability of individual features, such as “rapid fading”, to specific problems in language evolution studies (Galantucci et al. 2010); at a minimum, Hockett’s lists provide a useful historical yardstick. However, it is clear that overall, fruitful research into language evolution requires a distinctly non-Hockettian take.

## Footnotes

1 While “human language” may be viewed as pleonastic, we do not stipulatively tie the term language to humans, i.e. we believe the exclusion of non-humans is contingent and not definitional.

2 In many places, Hockett is straightforward about his theoretical affiliation; e.g. “… in this book, we shall deal with language in the frame of reference and the terminology of linguistics, rather than in those of anthropology, philosophy, psychology, foreign language teaching, or the like” (1958: 3).

3 For an interdisciplinary discussion of various definitions of communication, including Hockett’s, see Hauser 1996: 6–70.

4 We should note a logical error of comparing *communicative systems*, mapping signals/signs onto meanings (however defined), with *codes*, mapping symbols onto other symbols.

5 Explained in *Prolegomena to a Theory of Language* (Hjelmslev 1943).

6 The fifth communicative system included in the table is language, which by definition is endowed with all the properties.

7 Henceforth referred to as “Logical considerations” (1960b).

8 For the reasons he fails to explain from 1960’s “Origin of Speech,” Hockett uses the term “traditional transmission” instead of “cultural transmission.”

9 For an extensive discussion of the advantages and disadvantages of the vocal-auditory channel in comparison to the visual channel, see e.g. Fitch 2010.

10 Interestingly, the fact that the first attempts to teach chimpanzees human language focussed on speech (Gill 1997) also evidences a popular intuitive bias towards the vocal-auditory channel of communication.

11 This can be compared to a *nontrivial* similarity between bees and humans, that is underlying *cooperation*, which leads to a stable system of cheap but honest signalling (“conspiratorial whispers”, Krebs and Dawkins 1984) in the communication systems of both. Once more, accounting for it is impossible from a purely communicative perspective, but requires a broader socio-cognitive perspective informed by evolutionary theory and specifically, inclusive fitness.
